# Case study: Encouraging nurses to develop skills and confidence in Indonesia

**Published:** 2014

**Authors:** Widya Prasetyanti

**Affiliations:** Tells the story of Henny Nurjanah, a general nurse who has been working with Helen Keller International at Balai Kesehatan Mata Masyarakat, a community eye health centre in East Java, Indonesia, since 2010.

**Figure:**
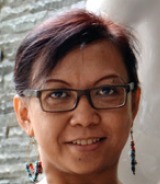
Widya Prasetyanti

As part of Henny's training in her new job, she was invited to take part in a training module on the early detection of eye disorders among children. Initially, she lacked confidence as she had never sat together with doctors and eye health experts before. She also felt she did not have experience outside of her daily duties as a nurse. However, she later attended a meeting of the module organisers where she gave input about how the module could be improved and her ideas were accepted, which pleased her.

Following an internship on a paediatric ward, she was invited to help develop a module on how to train others to screen children's eyes. The development team consisted of ophthalmologists, refractionists, opticians/optometrists, nurses and trainers from the provincial, city and regency departments of health, and was supported by child eye health specialists.

After several meetings, the team of ‘master trainers’ had to present the modules that they had developed. She had never expected to take the role of a master trainer. However, with encouragement from other members of the team, Henny presented a session to the others, who gave her feedback on how to improve her presentation skills.

Although she only had five people attending her first training session for other trainers, she felt very nervous. Over time, however, her confidence has grown. She has found that her experience – as an eye nurse who deals with children every day – strengthens her teaching, as it provides her with many practical examples of eye disorders she can share.

When she was asked if there were major changes in herself after becoming a trainer of trainers, Henny said: “The first time I delivered a training session, I prayed that none of the participants would ask questions. But now, it is me who prompts, ‘Is there anything you want to ask?’.” Her dealings with patients have also changed. “Now, my delivery and tone of voice are a bit different. I am more patient and more detailed when explaining something,” she said.

Henny has gained a lot by working at the eye health clinic for children. In addition to increasing her knowledge and making friends, she also gained the trust of her supervisor and colleagues in dealing with patients, particularly children. “If the intention is good, everything will go well, the main point is that I am happy working with children and collaborating with HKI,” she says.

**Figure:**
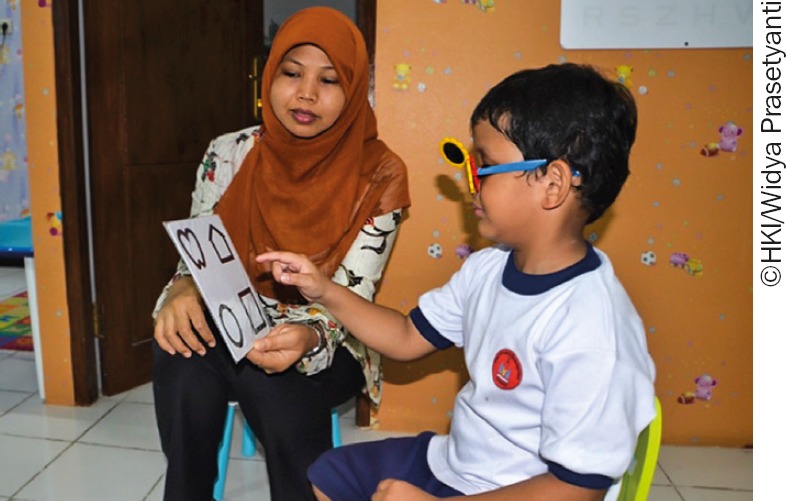
Henny Nurjanah uses Lea symbols to assess a child's vision

## Commentary

This case study highlights some important issues around involving nurses in the eye team, particularly when it comes to their professional development.

1. Nurses do not or automatically put themselves forward – they often lack confidence, perhaps because they are not traditionally included in planning or team meetings. As a result, nurses can remain under-valued and their considerable skills and experiences can remain under-utilised.
2. Building a team requires clear planning and direction – or key players could be omitted, as they will not put themselves forward.
3. With correct training and support, nurses are more confident and are valuable staff members who can deliver key work.
4. Involving all staff in training materials development not only improves the quality of the materials (because everyone brings additional experience and expertise), it can also help to improve self-confidence, create ownership and develop teams.
5. Individual confidence is built up over several events or training sessions – do not expect staff to gain confidence after one initial training event.
6. Given opportunities, most staff members will develop and grow. However, nurses in particular may need to be encouraged by their managers and colleagues until their own confidence develops sufficiently.

Written by Sally Crook

**FROM THE FIELD: Surgical nurses: key members of the operating theatre team**

**Figure:**
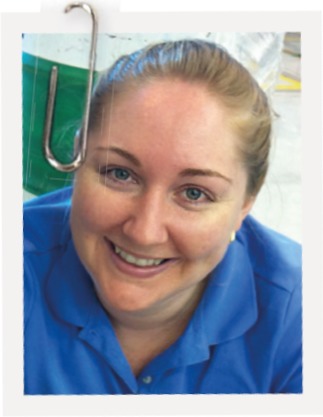
Heather Machin

**Heather Machin** is an ophthalmic and ambulatory surgery registered nurse who has worked in international development since 2007, and is a consultant with the Fred Hollows Foundation in New Zealand.

By working together as members of a functional and mutually supportive eye care team, we can provide high quality, patient-focused eye care. Nowhere is this more important than in the operating theatre, where the patient's life is in the hands of the health care providers.

Nurses are trained to focus on the needs and well being of the patient. If they are valued and respected by the other members of the surgical team, they will be able to speak out when they notice something that will put the patient's health or safety at risk. Not only will patient safety improve, but so too will the outcomes and patient satisfaction.

As an advocate for patient care, I am acutely aware that my role is to protect the patient from harm and to ensure a safe environment for the patient and colleagues. Throughout my career, I have had to speak out, sometimes in disagreement with others, to ensure policy and patient safety are adhered to at all times.

When I have a concern about equipment, procedures, timetabling, competence or the needs of the patient, I have a professional duty to speak out and inform others in the health care team.

This is also an essential component of the World Health Organization's Safe Site Surgery processes where individuals in the team are checking their environment, equipment, themselves and each other to ensure that patient care proceeds safely and as planned.

Nurses also have a personal responsibility to ensure they are reviewing their own work area, that they take part in any improvements, that they are participating in their own continual professional development and that they are maintaining knowledge of current policy at their workplace.

